# *In vitro* prion-like behaviour of TDP-43 in ALS

**DOI:** 10.1016/j.nbd.2016.08.007

**Published:** 2016-12

**Authors:** Phillip Smethurst, Jia Newcombe, Claire Troakes, Roberto Simone, Yun-Ru Chen, Rickie Patani, Katie Sidle

**Affiliations:** a Department of Molecular Neuroscience, UCL Institute of Neurology, Queen Square House, Queen Square, London WC1N 3BG, United Kingdom; b NeuroResource, UCL Institute of Neurology, Department of Neuroinflammation, 1 Wakefield Street, London WC1N 1PJ, United Kingdom; c London Neurodegenerative Diseases Brain Bank, Institute of Psychiatry, Psychology and Neuroscience, King's College London, DeCrispigny Park, London, United Kingdom; d Genomics Research Center, Academia Sinica, 128, Academia Road, Section 2, Nankang District, Taipei 115, Taiwan

**Keywords:** pTDP-43, phosphorylated TDP-43, IF, Immunofluorescence, WB, Western blotting, SS, Sarkosyl soluble, SI, Sarkosyl Insoluble, FL WT, Full length wild type TDP-43 plasmid, NC, Normal control, FCX, Frontal cortex, MC, Motor cortex, TCX, Temporal cortex, SC L, Spinal cord Lumbar, PDC, Parkinson's disease control, SK, Sarkosyl extract, NSC-34, Neuroblastoma spinal cord-34, MTT, 3-(4,5-dimethylthiazol-2-yl)-2,5-diphenyltetrazolium bromide, TDP-O, TDP-43 Oligomer, ALS, Prion-like disease, Protein misfolding, TDP-43, Seeding, Propagation

## Abstract

Amyotrophic lateral sclerosis (ALS) is the most common form of motor neuron disease (MND), and > 95% of familial and sporadic cases involve the deposition of insoluble aggregated, phosphorylated and cleaved TDP-43 protein. Accumulating clinical and biological evidence now indicates that ALS bears a number of similarities to the prion diseases, with TDP-43 acting as a misfolded ‘prion-like’ protein demonstrating similar underlying pathobiology. Here we systematically address the hypothesis that ALS is a prion-like disorder. First we demonstrate that TDP-43 demonstrates seeded polymerisation *in vitro* directly from both ALS brain and spinal cord. We next show that the seeding of TDP-43 results in the formation of characteristic insoluble, aggregated, and phosphorylated TDP-43 pathology that directly recapitulates the morphological diversity of TDP-43 inclusions detected in ALS patient CNS tissue. We next demonstrate that this reaction can be serially propagated to produce increasing amounts of phosphorylated TDP-43 pathology, and that aggregates can spread from cell to cell in an analogous fashion to that seen in the prion diseases. Finally, we reproduced our findings in a murine motor neuron-like cell line (NSC-34), where the seeding of TDP-43 induces the formation of TDP-43 oligomers and reduced cell viability. These findings may guide therapeutic strategies in this rapidly progressive and invariably fatal disease.

## Highlights

• Insoluble phosphorylated TDP-43 from ALS patient brain and spinal cord can seed into cell culture to form TDP-43 inclusions.
• These inclusions represent the full morphological spectrum of TDP-43 neuronal cytoplasmic inclusions observed in ALS.
• TDP-43 inclusions can be serially propagated resulting in an increase in phosphorylated TDP-43 pathology.
• TDP-43 aggregates can spread from cell to cell in a prion-like manner.
• TDP-43 can be seeded to motor neuron-like cells producing both TDP-43 oligomers and reduced cell viability.

## Graphical abstract

**Figure:**
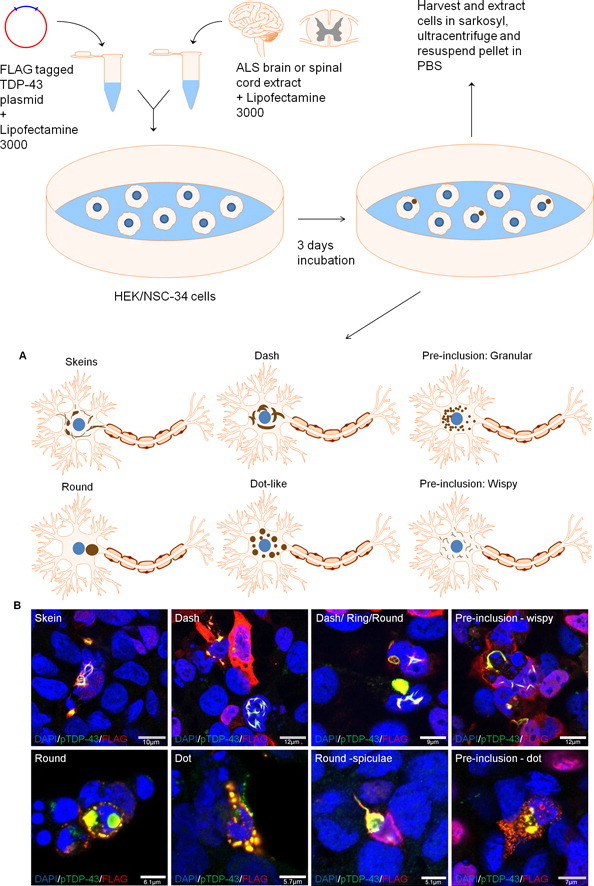

## 1 Introduction

Amyotrophic lateral sclerosis (ALS) is a progressive, age-related, fatal neurodegenerative disorder that predominantly causes the death of upper and lower motor neurons. This results in muscle spasticity, weakness, atrophy and eventual paralysis, where death is usually caused by degeneration of motor neurons innervating respiratory musculature (Wijesekera and Leigh, 2009). Accumulating evidence implicates a role for prion-like features in a number of neurodegenerative disorders including ALS (Frost and Diamond, 2010, Jucker and Walker, 2011, Polymenidou and Cleveland, 2011). Clinically, ALS demonstrates similarities to the prion diseases with a characteristic spreading of degeneration within the CNS. In ALS, this is often well recognised by a clear focal onset of clinical features which then spreads to neighbouring sites indicating disease propagation. (Ravits, 2014, Ravits et al., 2007a, Ravits and La Spada, 2009). Indeed, pathological examination corroborates clinical symptom spread, with a site of severe degeneration and cell loss from a focal onset in the neuraxis that diminishes further from the onset site (Ravits et al., 2007a, Ravits et al., 2007b).

The prion diseases are the prototypic protein misfolding disorders characterised by the accumulation of self propagating polymers of misfolded PrP. Cell to cell propagation of these misfolded protein polymers culminates in dissemination throughout the central nervous system (CNS). Different misfolded PrP subtypes, referred to as strains (Collinge et al., 1996), can lead to variation in both regional distribution and clinical features (Collinge and Clarke, 2007). It is currently not known how prions cause toxicity, however one hypothesis states that they may do so through the development of toxic oligomers (Sandberg et al., 2014, Sandberg et al., 2011) similar to that proposed with beta amyloid in Alzheimer's disease (Haass and Selkoe, 2007, Umeda et al., 2015, Wilcox et al., 2011). Indeed, this has led to an emerging theme among other neurodegenerative disease-related proteins including α-synuclein (Winner et al., 2011), Tau (Lasagna-Reeves et al., 2011, Tian et al., 2013), and now TDP-43 (Fang et al., 2014).

The pathological hallmark of ALS is the deposition of ubiquitinated aggregated proteins which, in > 95% of all cases, contain TDP-43 (Arai et al., 2006, Neumann et al., 2006). TDP-43 is coded for by the TARDBP gene on chromosome 1; it is a ubiquitously expressed and well conserved 414 amino acid protein. TDP-43 consists of an N-terminal domain, two RNA binding domains, a glycine-rich C-terminal and a bipartite nuclear export and localization sequence. TDP-43 is a multifunctional RNA binding protein normally localised in the nucleus, however in the majority of ALS cases it is mislocalised from the nucleus to the cytoplasm, where it becomes insoluble, hyperphosphorylated and cleaved (Arai et al., 2006, Hasegawa et al., 2008, Neumann et al., 2006). These pathological insights, taken together with the discovery that mutations in TDP-43 can cause ALS (Daoud et al., 2009, Kabashi et al., 2008, Sreedharan et al., 2008) and FTLD (Benajiba et al., 2009, Borroni et al., 2009), implicate TDP-43 as a pivotal pathogenic factor both in ALS and FTLD, with these forming an ALS/FTLD disease continuum (Janssens and Van Broeckhoven, 2013).

TDP-43 demonstrates significant prion-like features (reviewed in Smethurst et al., 2015). Recombinant TDP-43 can form aggregates *via* a proposed templated seeding reaction in cell culture (Furukawa et al., 2011), and TDP-43 aggregates from ALS and FTLD brains can also seed characteristic TDP-43 pathology in cell culture (Nonaka et al., 2013). TDP-43 seeded directly from human CNS tissue induces cellular toxicity, proteasome dysfunction, and cell-to-cell propagation (Nonaka et al., 2013), whilst demonstrating resistance to heat, denaturation and proteases. Pathological reports of phosphorylated TDP-43 (pTDP-43) in ALS post-mortem brain (Brettschneider et al., 2013) and spinal cord (Brettschneider et al., 2014) suggest that TDP-43 pathology could spread *via* axonal trajectories and synaptic connections as well as non-contiguous spread. Further, the majority of pathogenic ALS-causing TARDBP mutations occur in the C-terminus of TDP-43 (Pesiridis et al., 2009), which is involved in protein-protein interactions (Kuo et al., 2009) and protein localisation (Nonaka et al., 2009b). The C-terminus has been termed a ‘prion-like’ domain as it contains a glutamine and asparganine (Q/N) rich region which regulates its self association, aggregation and amyloid forming potential (Fuentealba et al., 2010, Saini and Chauhan, 2014, Saini and Chauhan, 2011).

Against this background, it is possible that the prion-like behaviour of TDP-43 is a key mechanistic feature in ALS. Here, we use an integrated approach: isolating protein extracts from human post-mortem tissue and systematically transfecting them into different *in vitro* cellular model systems to investigate TDP-43 seeding potential and characteristics. We demonstrate that pathological pTDP-43 aggregates can be extracted from both ALS brain and spinal cord tissue, the latter arguably representing the more pathologically relevant tissue with respect to ALS, and form a seeded polymerisation in cell culture. These pTDP-43 assemblies can mislocalise endogenous TDP-43 from the nucleus to the cytoplasm where it becomes insoluble and aggregated; thus recapitulating the pathological hallmark of ALS, including a diverse array of established TDP-43 inclusion morphologies. In turn, we have demonstrated that this reaction can be serially passaged from cells containing pTDP-43 aggregates, and further increasing the amount of pTDP-43 pathology upon serial passage. We also demonstrate that these aggregates can spread from cell to cell in a prion-like manner. Finally, we show this seeding reaction in a motor neuron-like cell line that demonstrates formation of TDP-43 oligomers and significantly reduced cell viability.

## 2 Materials and methods

### 2.1 CNS tissue homogenate extractions

Post-mortem frozen CNS tissue samples were obtained from a number of sources including the NeuroResource tissue bank (UCL Institute of Neurology, London), the London Neurodegenerative Diseases Brain Bank (King's College, London) and the Queen Square Brain Bank for Neurological Disorders (UCL Institute of Neurology, London). All tissue samples were obtained with informed consent and under Research Ethics Commitee and Human Tissue Authority (HTA) regulations. ALS cases were clinically, electrophysiologically and pathologically definite cases. All brains were pathologically confirmed to have TDP-43 positive neuronal cytoplasmic inclusions.

To prepare phosphate-buffered saline (PBS) tissue extractions, each block of snap-frozen tissue was homogenised in 9 x tissue wet weight of PBS using a probe sonicator and 750ul aliquots were frozen at − 80 °C. For sarkosyl insoluble urea soluble fractions, CNS tissue was homogenised and extracted as described previously (Arai et al., 2006, Hasegawa et al., 2008) but with some modifications. For each extraction we took 500 μl of 10% tissue homogenate and added an equal amount of 2 × extraction buffer A (BA: 10 mM Tris, 0.8 M NaCl, 10% sucrose, 1 mM EGTA, pH 7.5) with 2% triton X-100, 2 × protease (Roche, Cat. no. 05892970001) and phosphatase inhibitor cocktail tablets (Roche, Cat. no. 04906837001). This was incubated for 30 min at 37 °C and ultracentrifuged (100,000 ×* g*, 30 min, 4 °C). The supernatant was discarded, and the pellet was briefly sonicated in the starting volume (500 μl) of 1 × BA containing 1% sarkosyl (Sigma, Cat. no. L9150) with 1 × protease and phosphatase inhibitors, then incubated at 37 °C for 30 min. The sample produced was ultracentrifuged (100,000 × g, 30 min, 23 °C) and the supernatant was discarded. The pellet was briefly sonicated in 200 μl of 1 x BA containing 1% CHAPS (Sigma, Cat. no. C9246) with the protease and phosphatase inhibitors, and ultracentrifuged for 20 min at 25 °C. The supernatant was removed and discarded and the pellet was resuspended 50 μl of 8 M urea (pH 7.5).

For the TDP-43 seeding the homogenate was extracted using the tissue homogenisation buffer (HB) made at 2 × concentration (HB: 10 mM Tris- HCl, pH 7.5 containing 0.8 M NaCl, 1 mM EGTA, 1 mM dithiothreitol) containing a 2% sarkosyl detergent and protease and phosphatase inhibitors. Then 750 μl of this buffer was added to the homogenates to make a final of 1 × HB with 1% sarkosyl. These samples were incubated at 37 °C for 30 min and centrifuged (12,000 ×* g*, 10 min). The supernatant was ultracentrifuged (50,000 ×* g*, 10 min, room temperature). The pellet was washed in sterile PBS, sonicated and re-ultracentrifuged (100,000 ×* g*, 20 min, room temperature). The final pellet was resuspended by sonication in 25 μl of sterile PBS per pellet. In order to achieve workable amounts of TDP-43 seeding, 4 to 6 aliquots of homogenate were extracted per CNS region.

### 2.2 TDP-43 constructs

The wild type FLAG tagged TDP-43 gene was previously cloned into the pFLAG-CMV2 vector which was a kind gift from Burratti and Barralle (Ayala et al., 2008). The full length wild type TDP-43 was transformed into *E. coli* to propagate the plasmid. For this 50-100 μl of High Efficiency JM109 or HB101 *E. coli* cells (thawed slowly on ice) and 5 μl–10 μl of ligation reaction (*i.e.* 10% of the cell volume) were mixed and incubated on ice for 15 min then “heat shocked” for 30 s in a 42 °C heating block and returned to the ice for 10 min. After the addition of 500 μl–1 ml of LB-broth (for low or high copy number vectors respectively), the cells were incubated at 37 °C for 1 h with horizontal agitation at 150 rpm. Gentle centrifugation at 3000 ×* g* formed a cell pellet which, after removal of the supernatant, was re-suspended in 100 μl of LB-broth. The full cell suspension was spread on an LB-agar plate containing an appropriate selection antibiotic and incubated overnight at 37 °C.

Single, well-defined colonies were individually picked and cultured in 3 ml of L-broth containing the selection antibiotic. Following overnight incubation at 37 °C with vigorous horizontal agitation at 250 rpm, the cultured cells were harvested by centrifugation at 17,000 ×* g*. Cloned plasmid DNA was extracted from the bacterial cells using the QIAquick Spin Miniprep kit (Cat. no. 12,123, QIAGEN) according to the manufacturer's protocol. For larger quantities of DNA the bacteria were grown in 250 ml of LB broth and incubated overnight at 37 °C with agitation at 250 rpm. After this, the QIAGEN maxi prep kit (Cat. no. 12362, QIAGEN) was used to extract and purify the DNA used for transfection according to the manufacturer's protocol. Final confirmation of cloned plasmid constructs was achieved by sequencing. All DNA sequencing was outsourced to a service provider, Source BioScience Ltd.

### 2.3 Cell culture

Human embryonic kidney (HEK) 293 and Neuroblastoma spinal cord 34 (NSC-34) cells were cultured in DMEM with high glucose and 5 mM Glutamine containing 10% FBS. Cells were grown at 37 °C and 5% CO_2_ up to 80–90% confluency then were passaged at 1 in 10 and grown for up to a week where the medium was changed every 2–3 days.

### 2.4 Transfection of plasmids and proteins to cell culture

Cells were either grown in 24 well plates for IF staining or 6 well plates for western blotting. In each case transfection of plasmids was performed using Lipofectamine 3000 (Invitrogen) according to the manufacturer's instructions. In brief, we used DMEM with no FBS as transfection media and added 0.75 μl of lipofectamine and 1 μl of P3000 reagent with 0.5 μg of plasmid DNA per 1 well of a 24 well plate, and 3.75 μl of lipofectamine with 5 μl of P3000 reagent and 2.5 μg of plasmid DNA per 1 well of a 6 well plate.

To inoculate the insoluble TDP-43 protein seed from the ALS CNS homogenates, the transfection process was the same as above but with 5 μg of protein in replacement of the DNA. Both plasmids and TDP-43 seeds were co-transfected and were incubated for a maximum of 3 days post transfection. For IF analysis the cells were fixed in 4% PFA. For western blotting and serial propagation the cells were harvested by trypsinising in 0.25% Trypsin with EDTA and centrifuged at 2000 rpm for 5 min. The media was discarded and the pellet was resuspended and sonicated in 50 μl of TS buffer (150 mM Tris HCl, 50 mM NaCl, 5 mM EDTA, and 5 mM EGTA) containing 1% sarkosyl and protease and phosphatase inhibitors. The samples were sonicated and incubated at 37 °C for 30 min. The samples were ultra-centrifuged at 290,000 ×* g* for 20 min at room temperature. The supernatant was used as the sarkosyl soluble (SS) fraction and the pellet was resuspended by sonication in either 25 μl of 8 M urea per 1 well of a 6 well plate for western blotting, or 25 μl of sterile PBS per 1 well of a 6 well plate for serial propagation. A BCA assay was performed on both fractions, and 10–20 μg of protein was loaded on a gel for blotting and 5 μg of protein from the PBS suspended pellet was transfected to the next culture.

### 2.5 TDP-43 propagation

For serial propagation 5 μg of the sterile PBS fractions of the treated cells were co transfected with the FL WT TDP-43 plasmid and incubated for 3 days and harvested as before where half of the pellet was used for western blotting and the other half was resuspended in sterile PBS for further propagation.

For aggregate spreading, HEK cells were grown to ~ 60–70% confluency in 24 well plates and either seeded with the FL WT and ALS inocula or transfected with GFP. After 3 days of incubation both wells were trypsinised in 0.25% trypsin for 5 min and spun down at 2000 rpm for 5 min. Both populations of cells were mixed together in a 1:1 ratio and plated onto a 22 mm diameter coverslip and incubated for a further 3 days. Cells were fixed in 4% paraformaldehye (PFA) for immunocytochemical analysis.

### 2.6 Immunocytochemistry

Cells were gently washed with PBS and fixed for 15 min in 4% PFA then gently washed again in PBS and permeabilised with 0.5% triton X-100 in PBS for 10–15 min. The coverslips were blocked in 5% BSA for 30 min and stained with either polyclonal rabbit *anti*-TDP-43 (1:500) (Proteintech, Cat. no. 10782-2-AP), monoclonal mouse *anti*-TDP-43 (1:500) (Proteintech, Cat. no. 60019-2-Ig), polyclonal anti-rabbit phospho-TDP-43 (pS409/410-1) (1:500) (CosmoBio, Cat. no. TIP-PTD-P01), polyclonal *anti*-rabbit TDP-O (1:500) (a gift from Dr. Yun Ru Chen), monoclonal anti-mouse FLAG M2 (Sigma, Cat. no. F3165) in 5% BSA for 2 h at room temperature. Cells were gently washed in PBS 3 times and incubated with fluorescent secondary anti-rabbit Alexa-Fluor 488 and anti-mouse or anti-rabbit Alexa-Fluor 568 antibodies (1:2000) (Invitrogen) in 5% BSA for 1 h and washed again as before. They were mounted with DAPI Fluoromount G onto slides, allowed to dry and visualised with a Zeiss 700 confocal microscope.

### 2.7 Immunoblotting

Samples were measured for protein concentration using a BCA assay (Bio—Rad) and either 10 or 20 μg of protein was loaded on to a gel. These samples were added to an equal amount of working SDS solution with β-mercaptoethanol and boiled at 100 °C for 5 min. The samples were vortexed and centrifuged at 14,800 rpm for 1 min. Equal amounts of protein from each sample were loaded on to a 15 well 16% tris glycine SDS PAGE gels (Cat. No. EC64985BOX, Invitrogen) and run at 200 V for 70 min. Gels were transferred and blotted onto nitrocellulose membranes (Cat. No. 10401196, Protran) for either 2 h at 35 V or 15 V at 4 °C overnight. Membranes were then blocked in 5% non-fat milk in PBS for 30 min and probed with either anti-rabbit polyclonal pTDP-43 (pS409/410) (Proteintech, Cat. No. 223091-AP) (1:1000), monoclonal anti-mouse FLAG M2 (Sigma, Cat. no. F3165) or mouse monoclonal β-actin antibody Clone AC-15 (1:20,000) (Cat. no. A1978, Invitrogen), in 1% non-fat milk in PBS + 0.1% tween (PBST). Blots were washed 3 times for 5 min each in PBST and probed with secondary fluorescent antibodies: goat anti rabbit IgG antibody IRDye800 (green) (Cat. no. 611-132-122, Tebu-bio) (1:10,000), Alexa Fluor 700 goat anti mouse IgG (red) (Cat. no. A-21036, Invitrogen) (1:10,000) in 1% Non-fat milk in PBST. Blots were washed again 3 times for 5 min in PBST and washed once in PBS, then visualised with an Odyssey scanner.

### 2.8 Cellular toxicity assays

For the assessment of cellular viability the 3-(4,5-dimethylthiazol-2-yl)-2,5-diphenyl- 2H-tetrazolium bromide (MTT) assay was performed using MTT salt (Sigma, Cat. no. M5655) resuspended at 1 mg/ml in sterile water. Cells were plated out in 96 well plates at 500 cells per well and co-transfected as previously described, then on day 3 20 μl of the 1 mg/ml MTT solution was added to each well and left to incubate at 37 °C for 2–4 h The media from each well was decanted and replaced with 100 μl of dimethyl sulfoxide (DMSO) until all the purple formazan product was dissolved and the plate was read at 570 nm. Absorbance readings were taken as a percentage of the mean value of non-treated cells.

### 2.9 Densitometry and statistical analysis

Densitometry analysis was performed using the raw data on the inbuilt Odyssey densitometry software and statistical analysis was carried out using Prism 4 software. An unpaired two tailed student's *t*-test was used when comparing between two individual groups cases to generate a statistical *p* value. A one way ANOVA was used when comparing 2 or more groups with a *post hoc* Tukey test to compare all groups. Any p value below 0.05 was considered to be statistically significant (*p < 0.05, **p < 0.01, ***p < 0.001).

## 3 Results

### 3.1 Seeding and aggregation of TDP-43 from ALS CNS tissue

**Fig. 1:**
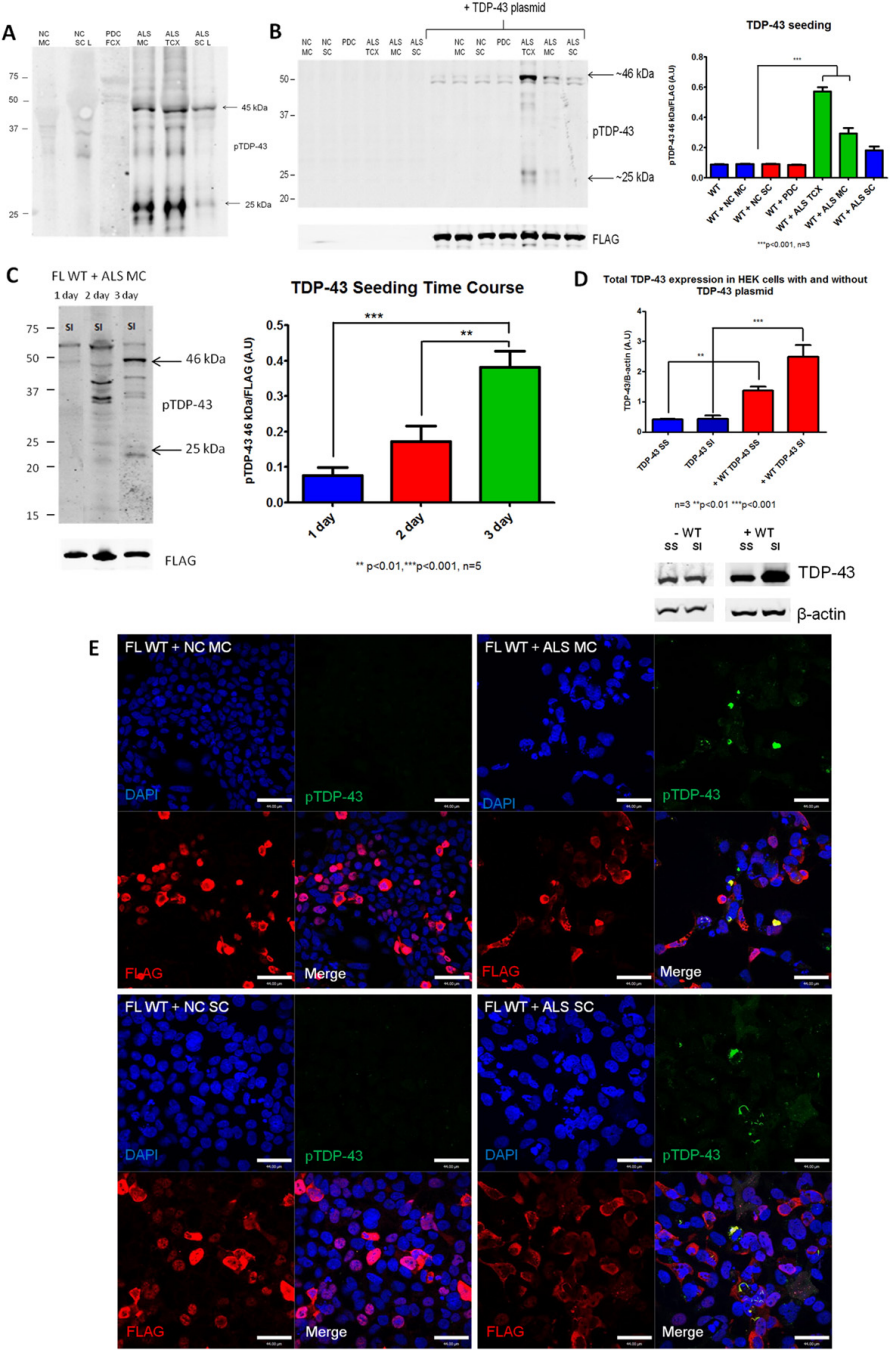
**Seeded aggregation of pTDP-43 from ALS brain and spinal cord.A)** Western blotting of Sarkosyl insoluble, urea soluble control and ALS patient samples which were used as pTDP-43 enriched inocula, labelled using polyclonal *anti*-pTDP-43 (pS409/410) antibody. **B)** Western blotting of Sarkosyl insoluble (SI) fractions of HEK cells either without plasmid or expressing the FL WT TDP-43 plasmid. The cells received either no inocula or 5 μg of normal control (NC), parkinson's disease control (PDC) or ALS inocula from motor cortex (MC), temporal cortex (TCX), or spinal cord (SC). Western blots labelled using anti-FLAG and anti-pTDP-43 antibodies. The graph shows the densitometry of pTDP-43 46 kDa band from SI fractions of cells expressing WT TDP-43 treated with control or ALS inocula (*n* = 3 and **p < 0.01) **C)** Sarkosyl insoluble (SI) fractions of HEK cells transfected with the FL WT TDP-43 plus 5 μg of ALS inocula from ALS MC at 1, 2 and 3 days post inoculation and western blotted using monoclonal anti-FLAG and polyclonal anti-pTDP-43 antibodies. The pTDP-43 bands are indicated with arrows, and quantification was performed on the 46 kDa band using densitometry from *n* = 5. **D)** Quantification of endogenous TDP-43 levels from SS and SI fractions of HEK cells expressing no construct (blue) and expressing the FL WT construct (red). Blots are labelled with polyclonal TDP-43 and β-actin of cells either without TDP-43 plasmid (−) or expressing the TDP-43 plasmid (+), and are representative of *n* = 3. **E)** Immunofluoresence (IF) staining of HEK cells transfected with 5 μg control or ALS pTDP-43 MC and SC enriched inocula with the FL WT plasmid. Scale bars = 44 μm, NC = normal control, MC = motor cortex, TCX = temporal cortex, PDC = Parkinson's disease control, SS = sarkosyl soluble, SI = sarkosyl insoluble, FL WT = full length wild type TDP-43 plasmid. All blots and IF images are representative images of n = 3. All western blots were loaded with equal amounts of protein in each well per gel. Error bars represent SEM. Statistical significance was calculated using an unpaired two tailed students *t*-test where **p < 0.01 and ***p < 0.001.

To test the hypothesis that a TDP-43 seeding reaction can be initiated from both brain and spinal cord ALS post-mortem tissue into cell culture, we extracted protein lysates from ALS, pathologically confirmed, brain and spinal cord samples. On repeated testing, only samples containing phosphorylated TDP-43 on Western blotting (WB) were able to seed TDP-43 aggregation. Therefore, samples exhibiting robust phosphorylated TDP-43 (pS409/410 or ‘pTDP-43’) pathology were selected for further experiments (Fig. 1A and Table S1). In order to test the conditions necessary to seed TDP-43 in cell culture, we transfected 5 μg of protein extract from control or ALS cases into HEK293 cells which were either unmodified or expressed a full length wild type TDP-43 FLAG-tagged construct. Immunofluorescence (IF) labelling of cells or western blot (WB) analysis on the sarkosyl insoluble (SI) cell fractions was next conducted (soluble fractions demonstrated no changes or development of pTDP-43 pathology; data not shown). Sarkosyl insoluble extracted samples from normal control brain, spinal cord and Parkinson's disease brain were used as healthy and disease controls respectively (Fig. 1A). Transfection of pTDP-43 extracts from ALS brain or spinal cord alone into HEK cells did not reveal any pTDP-43 pathology after 3 days of incubation on a WB or immunocytochemistry (ICC) (Fig. 1B and E). This demonstrates two points; firstly that the ALS inocula alone is not enough to induce the formation of pTDP-43 inclusions in the absence of an overexpressed TDP-43 construct, and secondly, that any signal observed was not simply due to the presence of residual starting inocula material. In contrast, co-transfection of HEK cells with the full length WT construct (FL WT) and ALS brain or spinal cord samples, revealed pathological pTDP-43 bands on WB (Fig. 1B) after 3 days. The quantification of these bands demonstrates a significant increase in the 46 kDa band of pTDP-43 at 3 days post incubation with ALS temporal cortex (TCX) and motor cortex (MC) samples (***p < 0.001, *n* = 3) compared to the cells expressing the FL WT and FL WT seeded with control inocula. However, whilst the signal from the ALS spinal cord (SC) demonstrates an increase, this does not reach significance here (Fig. 1C). In this experimental condition alone, we observed the formation of numerous pTDP-43 positive cytoplasmic aggregates, which co-localised with the FL WT TDP-43, and depleted the nucleus of FL WT TDP-43 (Fig. 1E). This is reminiscent of TDP-43 pathology observed in CNS tissue from patients with ALS and FTLD. Indeed, the pTDP-43 inclusions formed from inoculation with ALS brain tissues more commonly formed the 25 kDa pTDP-43 bands, compared to the spinal cord samples which only formed the 45 kDa pTDP-43 band (appears as ~ 46 kDa due to the added 1 kDa FLAG), which is in agreement with previous observations of enrichment of the 25 kDa C-terminal fragments (CTFs) in ALS brain rather than spinal cord tissue (Igaz et al., 2008).

To ensure that the formation of pTDP-43 pathology observed from these experiments was a result of *de novo* propagation, and not persistence of the pTDP-43 from the transfected ALS inocula, a time-course seeding experiment was conducted over 3 days (Fig. 1C). These data demonstrate an increasing presence of pTDP-43 bands over 1, 2 and 3 days. Indeed, the densitometry of the pTDP-43 46 kDa band demonstrates significant increases between day 1 and 2 (***p < 0.001, *n* = 5) and day 2 and 3 (**p < 0.01, n = 5) (Fig. 1E). This is consistent with genuine *de novo* aggregation (Fig. 1C). Furthermore, in the ALS motor cortex treated cells, the 46 kDa band forms before the 25 kDa band from day 2 to 3 suggesting that the cleavage of TDP-43 occurs post phosphorylation (Fig. 1C), and the full length protein becomes phosphorylated first.

Noting that the TDP-43 seeding reaction is more efficient in cells expressing the full length TDP-43 wild type construct, we hypothesized that TDP-43 overexpression in these cells increases reaction efficiency (by increasing the substrate concentration). We measured the levels of sarkosyl soluble (SS) and insoluble (SI) TDP-43 on blots with densitometry, and confirmed a significantly increased levels of total TDP-43 in both SS and SI fractions in cells expressing the FL WT plasmid (‘+’) compared to the cells without the plasmid (‘–‘) (Fig. 1D; ***p < 0.001).

### 3.2 Morphological diversity of seeded TDP-43 inclusions

**Fig. S1:**
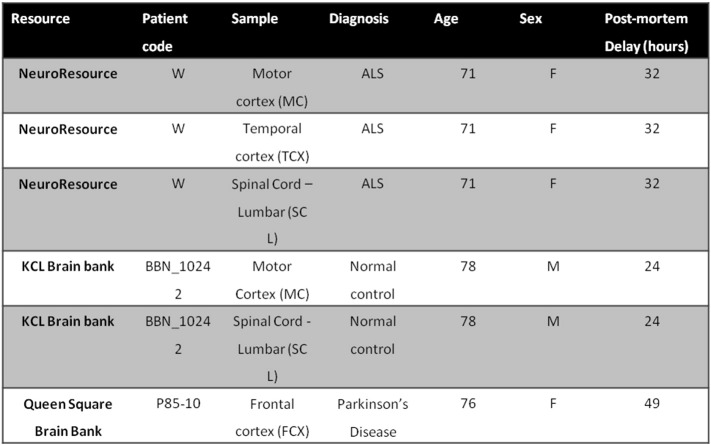
Morphological diversity of pTDP-43 inclusions seeded into HEK cells. A) and B) are skein-like pTDP-43 and FLAG positive inclusions. C) Mixtures of cytoplasmic pTDP-43 and FLAG positive dot and skein-like dash inclusions. D) Mixtures of dash, dense compact and ring inclusions positive for pTDP-43 and FLAG. E) Round pTDP-43 and FLAG positive inclusions. F) Round inclusion with radiating spiculae (racket shaped) positive for pTDP-43 and FLAG. G) Round pTDP-43 and FLAG positive inclusions with radiating spiculae. H) Circular pTDP-43 and FLAG positive inclusion. I) Dot like inclusions positive for FLAG and pTDP-43. J) Numerous dot inclusions positive for pTDP-43 and FLAG. K) Widespread dot inclusions throughout the cell positive for pTDP-43 and FLAG. L) Widespread dot inclusions positive for pTDP-43 and FLAG. M) Large dot inclusions positive for pTDP-43 and FLAG. N) Dot inclusions positive for TDP-43 and FLAG. O) Pre-inclusion with a mix of wispy straight and wavy filaments positive for pTDP-43 and FLAG. P) Diffuse granular pre-inclusion positive for pTDP-43 and FLAG with early coalescence into round inclusions. All images are representative images for the types of inclusions observed in each ALS sample examined. Cells were stained for DAPI (DNA/nuclear marker) in blue, pS409/410 (pTDP-43) in green, FLAG in red and all are images merged.

**Fig. 2:**
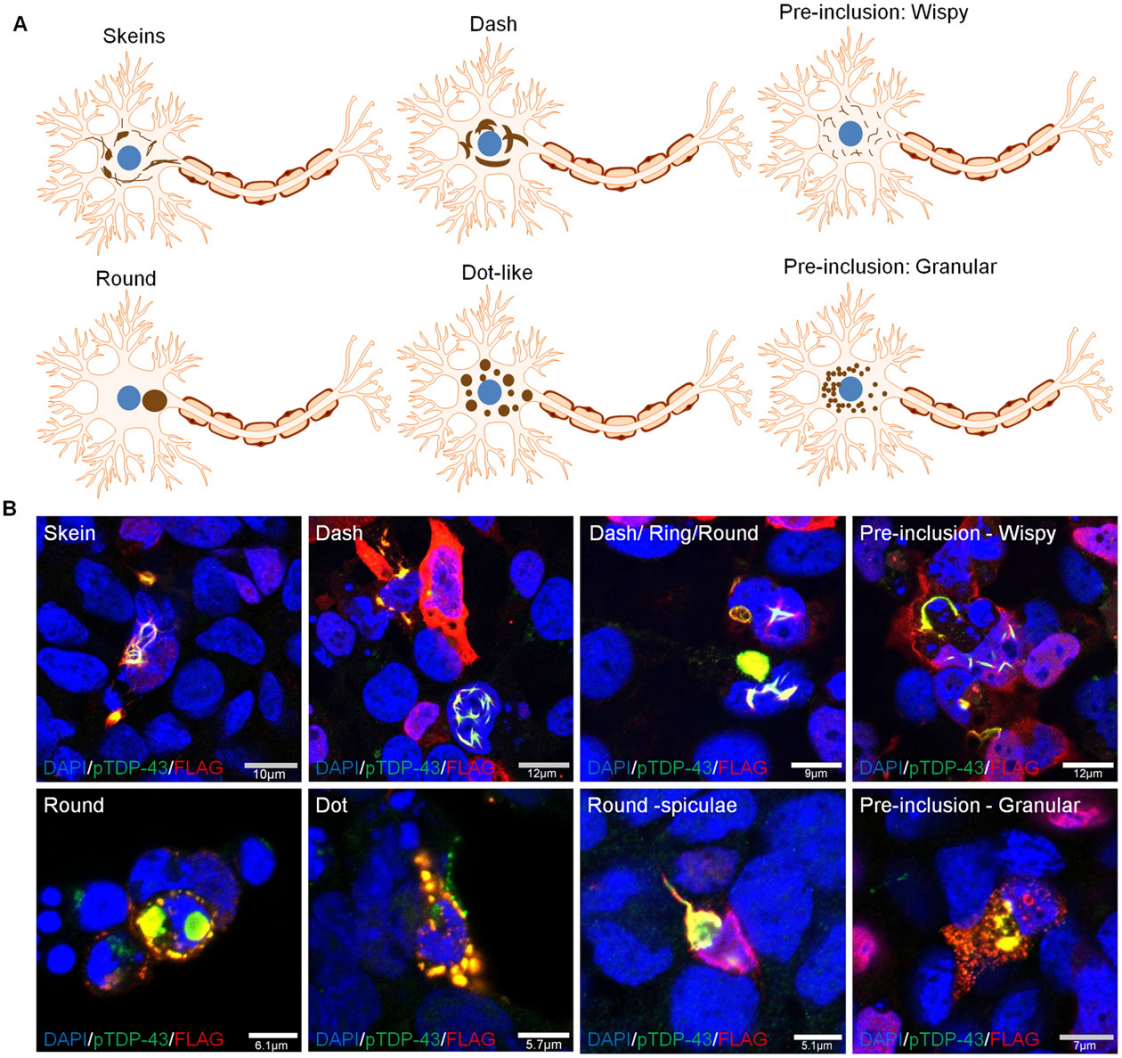
**Morphological diversity of pTDP-43 inclusions seeded into HEK cells.A)** Diagrammatic illustration of different morphological types of neuronal cytoplasmic inclusions seen in motor neuron cell bodies from patients with ALS. **B)** Different morphological types of inclusions observed upon seeding of pathological pTDP-43 (green) from ALS patient CNS tissue onto cells expressing FLAG tagged FL WT TDP-43 (red).

TDP-43 inclusions in the brain and spinal cord of patients with ALS can have varying morphologies including pre-inclusions (wispy and granular), skein, dash, dot, and round inclusions (Mori et al., 2008) (Fig. 2A). From our immunofluoresence staining experiments of cells seeded with TDP-43 from ALS brain or spinal cord, we confirmed that this full spectrum of morphological inclusion types are faithfully recapitulated in cell culture, and co-localise with FLAG tagged and endogenous TDP-43 (Figs. 2B and S1). Indeed, different morphologies can be present in close proximity to others (Fig. 2B, Dot/Dash/Round) suggesting a high degree of heterogeneity of inclusions occurring within the same sample. The co-localisation of the FLAG tagged TDP-43 in the aggregates is highly suggestive of a *de novo* seeded aggregation reaction. When the cells were stained for total TDP-43 the endogenous TDP-43 can also be seen to be removed from the cytoplasm and recruited in these aggregates alongside the FLAG tagged construct (Fig. S1N). These cells also demonstrate characteristic mislocalisation of TDP-43 (FLAG tagged and endogenous) from the nucleus to the cytoplasm (Fig. 2B) as seen in patients with ALS and FTLD. However, some inclusions are also formed with the FL WT TDP-43 still present in the nucleus (Figs. 2B and S1A), suggestive of an incomplete mislocalisation and aggregation process.

Additionally, the presence of diffuse cytoplasmic staining in the form of granular ‘pre-inclusions’ (Fig. 2B) suggests that some of these aggregates may still be in the maturation stage (Mori et al., 2008). Here we demonstrate the formation of two distinct types of diffuse cytoplasmic deposits: linear wisps and punctate granules, which occur in cells expressing the FL WT TDP-43 plasmid and treated with ALS inocula. Additionally, some round pTDP-43 inclusions can contain radiating spiculae in their margin, or fringes of thread like structures which are typical of inclusions observed in patients with ALS (Lowe et al., 1988, Murayama et al., 1990, Sasaki and Maruyama, 1991). These particular morphological characteristics can also be observed in the pTDP-43 inclusions formed here (Fig. 2B).

### 3.3 Serial passage of TDP-43 pathology

**Fig. 3:**
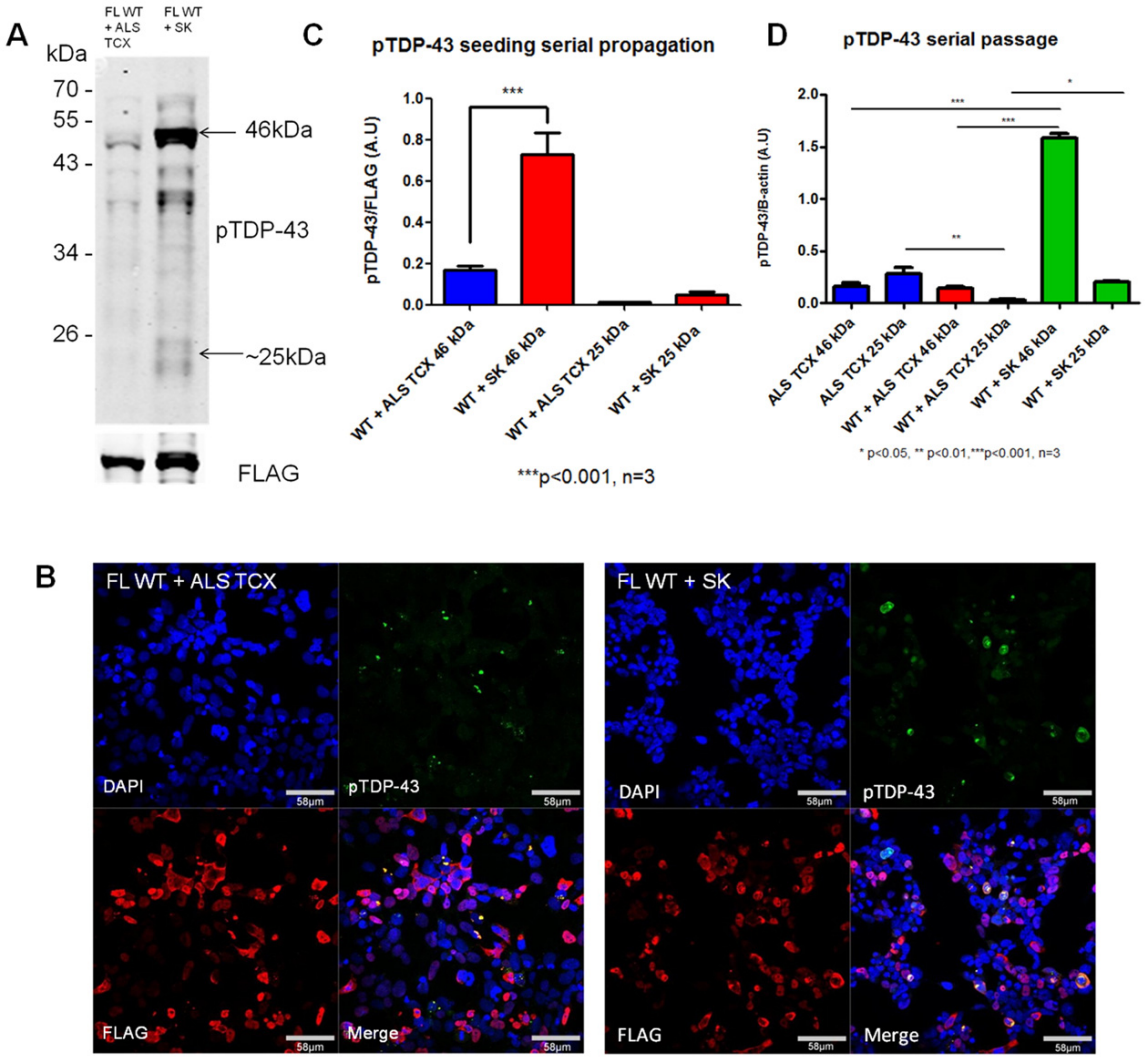
**Serial passage of pTDP-43 aggregates to naïve cells expressing TDP-43**. HEK cells containing aggregates that were co-transfected with full length wild type TDP-43 plasmid (FL WT) and ALS TCX were extracted in sarkosyl and the insoluble fraction was inoculated into naïve cells expressing FL WT TDP-43 in order to passage the seeding reaction. Western blot **(A)** and immunofluorescence **(B)** demonstrates the formation of pTDP-43 bands and aggregates with the FL WT + ALS TCX, and then stronger bands and aggregates in the FL WT + SK (insoluble fraction of HEK cells containing aggregates) after 3 days. Diagram **(C)** shows the increased amount of pTDP-43 46 kDa bands quantified by densitometry in passaged cells compared to cells exposed to initial ALS TCX extract, and corrected for FLAG expression. **D)** Densitometry of pTDP-43 46 and 25 kDa bands corrected for using β-actin in the initial inocula and the first and second passage of the cells. Error bars represent mean ± SEM where *n* = 3 and significance was determined using an unpaired two tailed students *t*-test (*p < 0.05, **p < 0.01,***p < 0.001).

Misfolded prion proteins representing different prion strains can be serially passaged *in vitro* and *in vivo* to maintain the strain specific conformational protein structures (Collinge and Clarke, 2007). We next investigated if we were able replicate this in cell culture by extracting the sarkosyl insoluble fraction from cells transfected with the FL WT TDP-43 plus ALS tissue extracted pTDP-43 (ALS TCX), and then re-transfecting of 5 μg of the cell extract (SK) into naive cells expressing FL WT TDP-43 (Fig. 3). We confirmed a pathological TDP-43 seeding reaction in the new cells using western blot and immunofluorescent staining (Fig. 3A and B). Despite ensuring that equal quantities of protein were loaded on the initial seeding reaction and passage, there was a significant increase in the amount of pTDP-43 46 kDa band on western blotting (***p < 0.001; Fig. 3C) in the cells exposed to passaged extract compared to cells exposed to the ALS TCX inocula. In order to determine if this result was simply due to increased amounts of seed in the inocula, or an enhanced seeding capacity, we corrected the levels of pTDP-43 bands with β-actin to determine the total levels of pTDP-43 in each treatment. Cells expressing the FL WT TDP-43 treated with ALS TCX inocula, in fact demonstrated lower levels of the pTDP-43 25 kDa band compared to the original ALS TCX inocula. However, after subsequent passage of inocula prepared from these cells onto naive cells expressing FL WT TDP-43, there was a significant increase in the pTDP-43 25 kDa bands (*p < 0.05; Fig. 3D). This increase in pTDP-43 levels following passage may therefore be consistent with a more efficient propagation, reminiscent of the adaption seen in prion passage into a new host.

### 3.4 TDP-43 aggregate spread

**Fig. 4:**
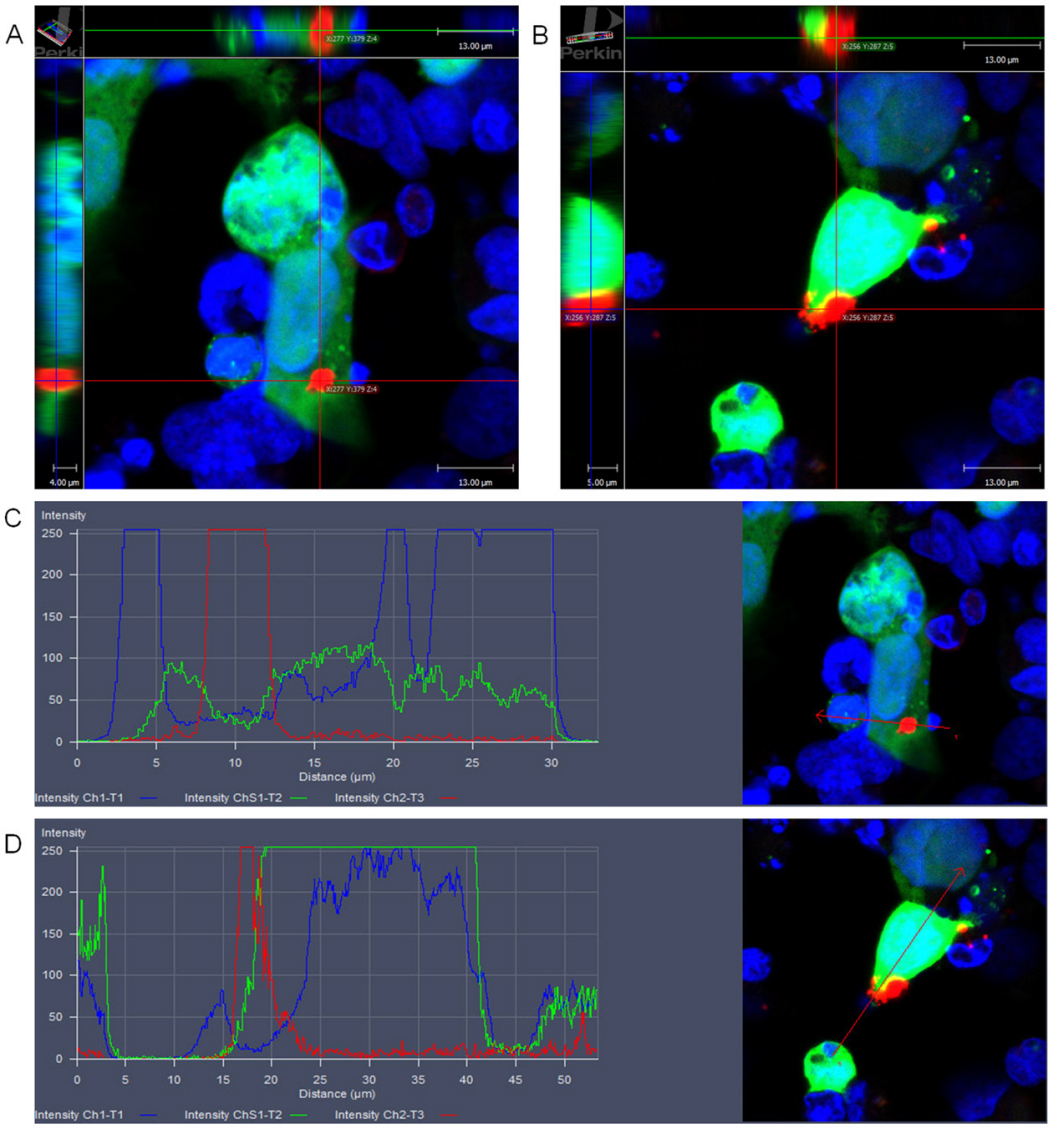
**pTDP-43 aggregate spread to naïve cells expressing GFP.** Co-culture of cells containing pTDP-43 aggregates and cells expressing GFP in a 1:1 ratio. After 3 days of incubation the cells were stained with pS409/410 (pTDP-43) (red) and DAPI (blue). Images **(A)** and **(B)** are 3D reconstructions of cells with pTDP-43 aggregates (Red). The red lines represent the X-Y axis, the green line represents the X-Z axes and blue line represents the Y-Z axes. Images **(C)** and **(D)** are intensity distribution profiles of images **(A)** and **(B)** respectively, with blue lines representing DAPI, green lines representing GFP and red line representing pTDP-43 in a merged image. All images are representative of 3 independent experiments.

Previous publications (Ding et al., 2015, Nonaka et al., 2013) have demonstrated that TDP-43 inclusions can spread from cell to cell in a prion-like manner although the mechanism of how this occurs remains unclear. In order to confirm these findings, we co-cultured cells containing pTDP-43 inclusions and mixed them in a 1:1 ratio with cells expressing GFP. The cells expressing GFP were used as markers of ‘acceptor cells’ or naïve cells for the potential uptake of aggregates in the propagation process. These cells were then further cultured for 3 days and immunostained for pTDP-43 (pS409/410) and analysed for the presence of pTDP-43 aggregates within cells expressing GFP. Fig. 4A and B are representative images of cells containing pathological cytoplasmic pTDP-43 inclusions which also express GFP. The 3D reconstruction of these images show the presence of the cytoplasmic inclusion in all axes (Fig. 4A and B), and the intensity distribution profiles show the presence of the pTDP-43 inclusion (red line) with GFP (green line) and its cytoplasmic localization (nucleus is the blue line) (Fig. 4C and D). These data indicate that the inclusions of pTDP-43 from the original seeded cells are now present in the acceptor cells, which further supports the occurrence of pTDP-43 aggregate spread from cell to cell.

After 3 days the number of cells expressing GFP and pTDP-43 inclusions were low, indicating that either the aggregate spreading may be a slow process, may be rate limited by the seed concentration, or that the non-neuronal cell type here may not provide a suitable architecture for the propagation process. As an additional method of exploring pTDP-43 aggregate propagation we also attempted to use conditioned media from cells treated with ALS seeds and incubate this conditioned media on fresh naïve cells for 3 days. In this case we were unable to observe the formation of any pTDP-43 inclusions in cells treated with the conditioned media.

### 3.5 TDP-43 seeding and toxicity in motor neuron-like cell line

**Fig. 5:**
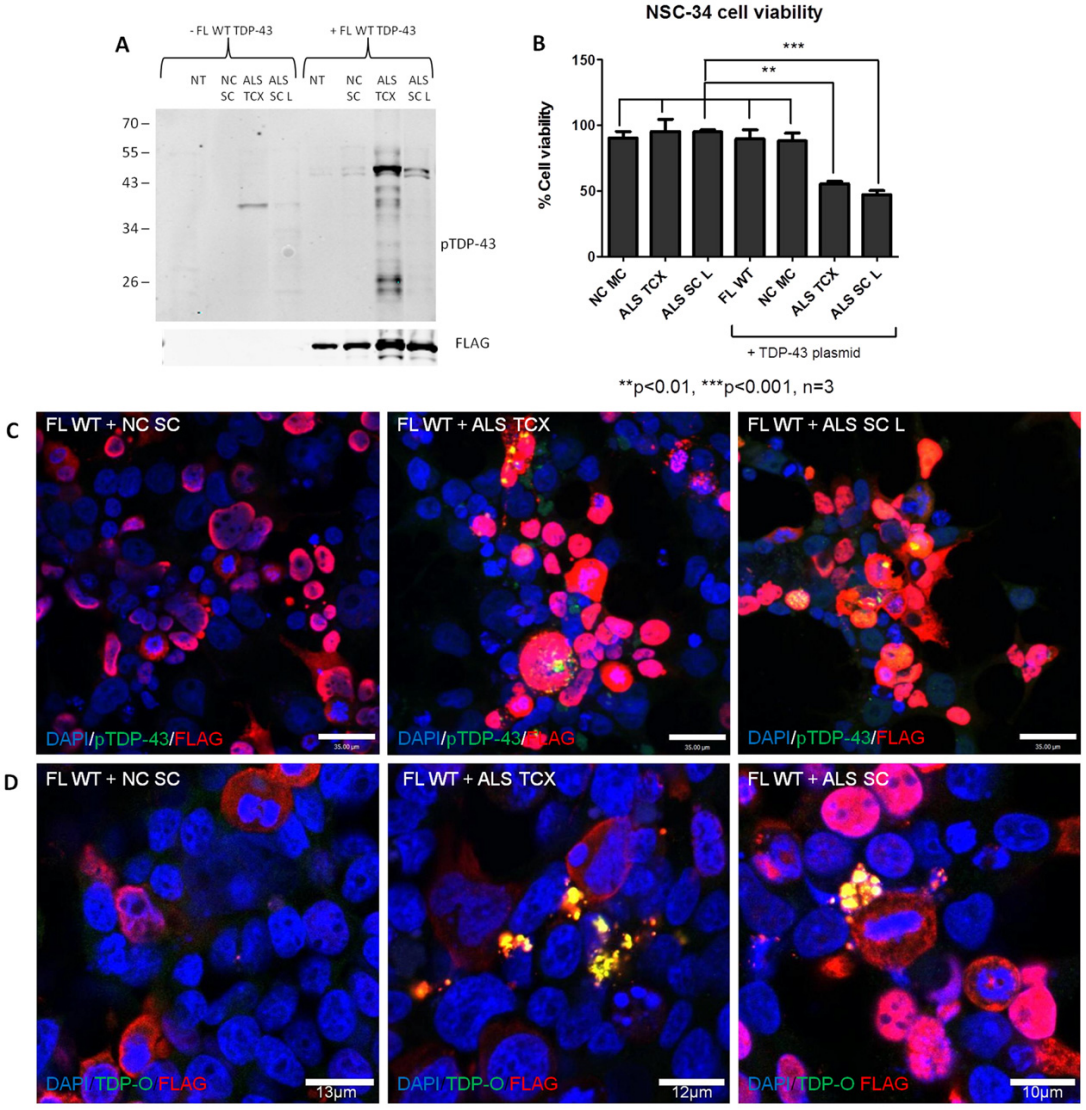
**TDP-43 seeding, toxicity and oligomer formation in a motor neuron like (NSC-34) cell line**. **A)** Western blot showing the formation of pTDP-43 46 and 25 kDa bands only when both the FL WT and ALS TCX and SC samples are transfected and not present in cells transfected with the ALS TCX or SC samples alone. **B)** MTT assay measuring NSC-34 cell viability after inoculation with control and ALS samples ± the FL WT TDP-43 plasmid showing significantly reduced cell viability only in cells inoculated with the plasmid and ALS samples compared to all other treatments. Error bars represent mean ± SEM with three repeats, and analysis was conducted by a one way ANOVA with a *post hoc* Tukey test to compare all groups. **p < 0.01 and ***p < 0.001. **C)** Representative images of the formation of pTDP-43 aggregates (green) in the NSC-34 cell line treated with ALS TCX and SC samples which are not present in control treated cells. All scale bars = 35 μm. **D)** Representative images of TDP-43 oligomers (TDP-O) (green) present in cells seeded with ALS samples and not controls.

In order to build a more relevant cellular model of TDP-43 seeded aggregation and toxicity, we replicated our original seeding experiments in a mouse motor neuron-like cell line. This cell line is a hybridoma fusion of mouse embryonic motor neuron enriched cells with mouse neuroblastoma cells producing the neuroblastoma spinal cord cell line (NSC-34). These cells are immortalized motor neurons that express many physiological characteristics of primary motor neurons (Cashman et al., 1992). In addition, they can also be differentiated to develop into mature cholinergic motor neurons as a useful model of motor neuron degeneration (Maier et al., 2013). Using this cell line, we next demonstrated that TDP-43 can be seeded directly from ALS CNS tissue extracts. We demonstrated that both ALS brain and spinal cord extracts can induce the formation of pTDP-43 bands at ~ 46 kDa and ~ 25 kDa after co transfection of the cells with the TDP-43 plasmid at 3 days (Fig. 5A). This was also demonstrated with pTDP-43 immunofluorescence staining of the NSC-34 cells whereby the only inclusions detected were in cells inoculated with the TDP-43 plasmid and the ALS brain or spinal cord extracts compared to cells transfected with the TDP-43 plasmid and control (Fig. 5C).

Next, to determine if the pTDP-43 aggregates were toxic to these cells, we performed a 3-(4,5-dimethylthiazol-2-yl)-2,5-diphenyltetrazolium bromide (MTT) assay to assess cellular viability. We treated the NSC-34 cells with a control spinal cord sample (NC SC) and separate CNS regions that we had previously demonstrated significant seeding capacity, which were the ALS brain (TCX) and spinal cord (SC) samples. The MTT assay revealed no reduction in cell viability in cells treated with the ALS brain and spinal cord extracts alone in comparison the control brain extract (Fig. 5B). However, cells expressing the TDP-43 plasmid and then treated with the ALS brain and spinal cord extracts, demonstrated significant reduction in cellular viability with brain (*p < 0.05) and spinal cord (**p < 0.01) compared to cells treated with TDP-43 plasmid alone and TDP-43 plasmid plus control (Fig. 5B). This would suggest that the reduced cell viability observed is due either to toxic effects of the aggregated TDP-43 or accelerated loss of normal function of TDP-43 in the seeded cells, or both.

Increasing evidence now implicates oligomeric species of aggregated proteins as the neurotoxic component in a number of neurodegenerative diseases (Colla et al., 2012, Fá et al., 2016, Fang et al., 2014, Lasagna-Reeves et al., 2011, Tian et al., 2013, Usenovic et al., 2015, Ward et al., 2012, Wilcox et al., 2011, Winner et al., 2011). Indeed, more recent evidence demonstrates the presence of toxic TDP-43 oligomers in patients with FTLD and ALS (Fang et al., 2014, Kao et al., 2015). To further demonstrate one of the potential causes of this observed reduced cell viability we wanted to determine the presence of these oligomers in our cell model. In order to do this we used a polyclonal TDP-43 oligomer (TDP-O) antibody developed by Dr. Yun-Ru Chen and colleagues (Fang et al., 2014). We used this TDP-O antibody to stain for TDP-43 oligomers in our cells treated with the TDP-43 plasmid plus a control or ALS brain and spinal cord tissue. We demonstrate the presence of the type of spherical TDP-43 oligomers detected by Fang and colleagues, which co-localise with FLAG present only in cells treated with FL WT TDP-43 plus ALS brain or spinal cord samples but not in control treated cells (Fig. 5D).

## 4 Discussion

Developing cellular models of TDP-43 protein aggregation has been the focus of many studies in order to understand the protein aggregation process and the molecular mechanisms underlying the disease process in ALS or FTLD (Dewey et al., 2012). To date studies have largely utilised the overexpression of mutant, expanded, deleted or truncated constructs and chemical modification to induce the aggregation of TDP-43 (Johnson et al., 2009, Nonaka et al., 2009a, Nonaka et al., 2009b, van Eersel et al., 2011, Wang et al., 2013). Whilst ALS brain tissue has been previously seeded (Nonaka et al., 2013), we have developed a TDP-43 aggregation model that can be reproduced directly from pTDP-43 extracts from both the brain and spinal cord of patients with ALS, the latter, arguably being the more relevant pathological tissue in ALS, and which has not been previously demonstrated. We have also shown that only CNS samples demonstrating pTDP-43 on western blotting are capable of being transmitted to our cell system. Previous models demonstrated the seeding of TDP-43 from recombinant protein which was not phosphorylated. This discrepancy may be due to the fact that TDP-43 is very prone to aggregate *in vitro* (Johnson et al., 2009), and recombinant protein aggregates may not represent the TDP-43 aggregated species found in ALS pathological tissue.

We have demonstrated that ALS spinal cord extracts form less of the 25 kDa C-terminal fragments on western blotting of the seeded cell extracts, compared with seeding from brain tissue and recapitulates the distribution observed in ALS patients (Igaz et al., 2008). Indeed, if these C-terminal fragments are more prone to develop in the brain, it would suggest that the cleavage product may not be as important for toxicity, as cell death is much more prominent in the spinal cord compared to brain in ALS patients. From our time course experiments we also demonstrate that the cleavage of TDP-43, which results in the formation of the 25 kDa pTDP-43 fragments, occurs post-phosphorylation of the full length protein and that phosphorylation is an earlier event in ALS pathogenesis.

Whilst we required overexpression of normal TDP43 in order to demonstrate transmission, we did not see spontaneous generation of pathological TDP-43 in cells not seeded with ALS tissue, supporting that TDP-43 aggregation was exclusively as a result of cell seeding. This further supports the prion-like nature of TDP-43, with the requirement of a sufficient substrate and seed, as observed when modelling prion disease (Prusiner et al., 1990, Tamgüney et al., 2006).

Our next observation was the novel demonstration of a diverse range of aggregated pTDP-43 morphologies recapitulating those seen in post-mortem ALS CNS tissue. TDP-43 inclusions take on the form of either skein, dash, round, dot or granular ‘pre-inclusions’ (Mori et al., 2008). The presence of ‘pre-inclusions’ in our cell model indicate that some pTDP-43 aggregates are still immature, suggesting that with longer incubation periods they will mature into larger inclusions. It is thought that fine pTDP-43 positive filaments and wisp inclusions eventually mature into coarse thick skein inclusions, and round inclusions arise from the maturation of punctate granules (Mori et al., 2008). Prion strains can be serially passaged and propagated *in vitro* and *in vivo* to next generations of animals or naïve cells where they replicate the same conformational protein structure (Collinge and Clarke, 2007). This is important because, if prion-like mechanisms do underlie disease pathogenesis in ALS, then stable strains or ‘clouds’ of TDP-43 assemblies may be responsible for the varied clinical presentation in ALS and stereotypical network spread of pathology. We were able to demonstrate the passage of pTDP-43 pathology to FL WT TDP-43 expressing naïve cells by extracting and utilizing the insoluble fraction of the ALS tissue seeded cells, containing pTDP-43 aggregates. On serial passage there was an increase in the quantity of pTDP-43 46 kDa bands on a western blot (***p < 0.001) suggesting that this seeding process is accelerated upon passaging. The increased accumulation of aggregated TDP-43 on serial passage may suggest more efficient propagation on passage and raises the possibility of the existence of strains of pathological TDP-43; a phenomena first demonstrated in the prion diseases, but more recently also demonstrated with alpha synuclein (Bousset et al., 2013, Guo et al., 2013, Peelaerts et al., 2015) and Tau (Sanders et al., 2014). Whilst the increase in pTDP-43 fractions upon serial passage may simply represent an enrichment of seeding material in the second cycle of inoculation, comparing the total amount of pTDP-43 across all inoculas would suggest that our findings may represent an increase in efficiency of seeded propagation through selection of more efficient seeding species/assemblies. Future investigation will shed further light on this matter and will include the effects of further serial passaging of pTDP-43.

Next, we investigated whether pTDP-43 aggregates could spread from cell to cell in a prion-like manner and has previously been reported (Nonaka et al., 2013). After co-culturing cells containing pTDP-43 aggregates with ‘acceptor’ cells expressing GFP in a 1:1 ratio for 3 days, numerous aggregates were formed, and a small number of cells expressing GFP, demonstrated cytoplasmic pTDP-43 aggregates, suggesting that these aggregates are indeed spreading to new cells. Longer co-culture incubation periods with stable GFP expressing cell lines may result in more effective propagation. However, from our results, it does suggest that the pTDP-43 aggregates do spread from cell to cell in a prion-like manner. The fact that we did not observe propagation with conditioned media could be due to the short incubation times, negligible pTDP-43 aggregates secreted into the conditioned media, or aggregate instability. Alternatively, the cells may require close contact for signalling mechanisms required for aggregate spread/uptake. Whilst these experimental data demonstrate uptake of aggregates from one cell to another, rather than *de novo* aggregation in the receptor cells, they support the non-cell autonomous spread of ALS pathology (Ilieva et al., 2009).

The presence of TDP-43 in the exosomal fractions of TDP-43 expressing cells treated with the ALS extract has been previously demonstrated which suggests that TDP-43 pathology is propagated at least partly *via* exosomes (Nonaka et al., 2013). Indeed, other findings from cerebrospinal fluid suggest that TDP-43 aggregates may spread *via* exosomes and tunnelling nanotubules (Ding et al., 2015). Additionally, recent studies utilizing staging of pTDP-43 pathology in the brain (Brettschneider et al., 2013) and spinal cord (Brettschneider et al., 2014) suggest that pTDP-43 pathology may propagate transynaptically in an anterograde fashion along corticofugal axonal projections (Braak et al., 2013). Indeed, this is supported by data from the same group showing significant diffusion tensor imaging (DTI) changes along the selected regions originally measured from the pathological staging (Kassubek et al., 2014). The collective data here supports our findings of a cell to cell spreading of pTDP-43 pathology.

The exact cellular mechanisms for pathological TDP-43 spread still remain elusive. Aside from exosome propagation of pTDP-43, other mechanisms of potential aggregate spread include free floating aggregates or seeds or nanotubule transmission as seen previously with prions (Gousset et al., 2009) and now recently observed with TDP-43 (Ding et al., 2015). Additionally, the method of seed uptake by the recipient cell has yet to be discovered. These potential methods of uptake could include direct penetration of the plasma membrane, fluid phase endocytosis, or receptor-mediated endocytosis. Indeed, SOD1 has recently been shown to propagate *via* exosomes and free floating aggregates and seeds which are taken up by lipid raft macropinocytosis (Grad et al., 2014, Münch et al., 2011). Uptake of TDP-43 could also potentially occur *via* various other protein dependent uptake mechanisms such as clathrin, caveolin and dynamin-mediated endocytosis. Our cell model of aggregation will allow investigation of the exact mechanisms of this propagation and may provide essential clues as to how to target these mechanisms in an attempt to therapeutically arrest TDP-43 propagation.

In order to determine the effects of TDP-43 seeding and aggregation from ALS CNS extracts within a neuronal cell line, we replicated this seeding reaction in the mouse motor neuron-like cell line (NSC-34). We report the successful demonstration of TDP-43 pathology in the NSC-34 cell line expressing the TDP-43 plasmid and following exposure to ALS CNS extract. We also demonstrate that TDP-43 seeding can decrease the viability of these NSC-34 cells. As this decrease in cellular viability only occurs with the TDP-43 plasmid and ALS CNS extract exposure, it is highly probable that TDP-43 is the responsible species.

It is still unclear if or how protein misfolding and aggregation is toxic to cells. One hypothesis is the formation of oligomeric aggregation intermediates. A number of neurodegenerative disease related proteins now demonstrate that the oligomeric species of misfolded aggregated proteins may be responsible for neurodegeneration in a number of disorders (Tian et al., 2013, Wilcox et al., 2011, Winner et al., 2011). Indeed, recent findings now suggest that TDP-43 oligomers may also be the toxic component present in patients with FTLD (Fang et al., 2014). Here we demonstrate that TDP-43 seeding in the NSC-34 cell line can develop spherical annular TDP-43 oligomers as reported by Fang and colleagues, raising the possibility that these species may also be responsible for the observed TDP-43 mediated reduction in cell viability.

## 5 Conclusion

Our findings here demonstrate that TDP-43 can seed the formation of TDP-43 aggregates *in vitro* directly from both ALS brain and spinal cord extracts. The morphology of the pathological TDP-43 inclusions is highly reminiscent of the TDP-43 pathology seen in the ALS patients, and all different morphologies of inclusions can be observed. We have also demonstrated that TDP-43 can propagate from cell to cell in co-culture experiments and can be serially passaged from cell extracts containing pTDP-43 aggregates. The increased accumulation of aggregated TDP-43 on serial passage may suggest more efficient propagation, and raises the possibility of the existence of strains of pathological TDP-43. Finally, we are able to induce this TDP-43 pathology in a pathologically relevant motor neuron-like cell line (NSC-34) and demonstrate decreased cellular viability and the formation of oligomeric species of TDP-43. These data further support the prion-like nature of TDP-43, confirming and extending accumulating evidence for the propagative nature of ALS. Indeed, our experimental paradigm could be used in *in vitro* assays to explore these prion-like mechanisms further and to screen for therapies to inhibit TDP-43 seeding, propagation and toxicity in order to potentially ameliorate this devastating condition.

The following are the supplementary data related to this article.

**Table S1:**
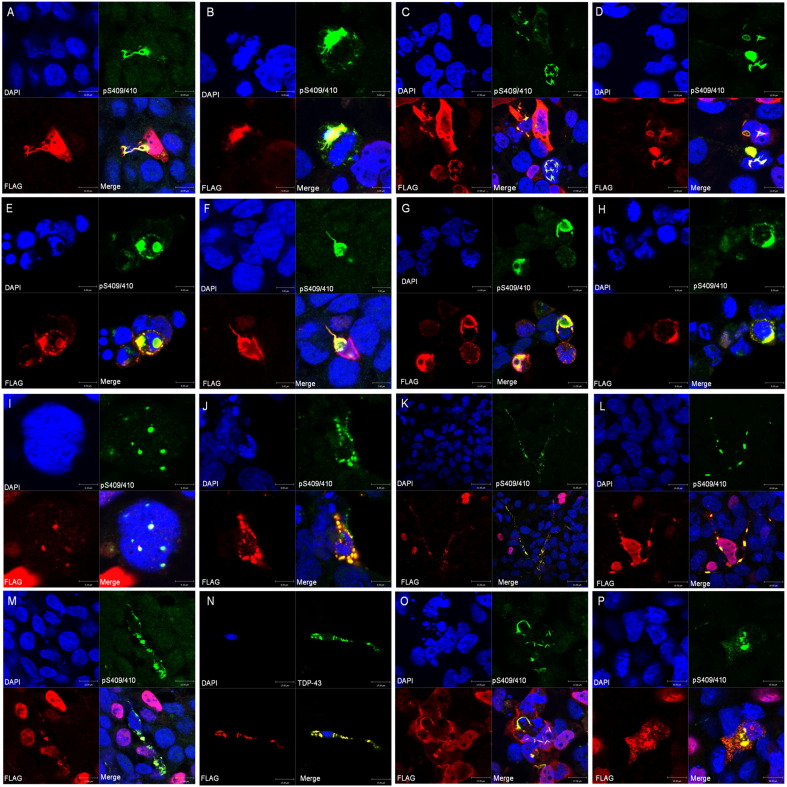
Control and ALS CNS tissue samples used in this study.
